# Field Evaluation of Novel Spatial Repellent Controlled Release Devices (CRDs) against Mosquitoes in an Outdoor Setting in the Northern Peruvian Amazon

**DOI:** 10.3390/tropicalmed7110372

**Published:** 2022-11-12

**Authors:** Carmen Flores-Mendoza, Victor M. López-Sifuentes, Gissella M. Vásquez, Craig A. Stoops, Michael L. Fisher, Ulrich R. Bernier, Melynda Perry, Juan Mollica, Damián A. Coltzau, Pablo Gurman, Sebastián D’hers, Noel M. Elman

**Affiliations:** 1 U.S. Naval Medical Research Unit No. 6 (NAMRU-6), Venezuela Ave, Block 36, Bellavista, Callao, Peru; 2 Center for Medical, Agricultural and Veterinary Entomology (CMAVE), United States Department of Agriculture-Agricultural Research Service (USDA ARS), 1600 SW 23rd Drive, Gainesville, FL 32608, USA; 3 The U.S. Army Combat Capabilities Development Command Soldier Center (DEVCOM SC), 15 General Greene Avenue, Natick, MA 01760, USA; 4 Computational Mechanics Center, Instituto Tecnológico de Buenos Aires (ITBA), Av. Madero 399, Ciudad Autónoma de Buenos Aires C1106ACD, Argentina; 5 GearJump Technologies, LLC, P.O. Box 1600, Boston, MA 02446, USA

**Keywords:** mosquitoes, *Anopheles*, Peru, controlled release device, spatial repellent, metofluthrin, malaria

## Abstract

U.S. military troops are exposed to mosquito-borne pathogens when deployed to endemic regions. Personal protective measures such as permethrin-treated uniforms and dermal repellents are the cornerstones of mosquito-borne disease prevention for the U.S. military. These measures have limitations and additional personal protection tools, such as spatial repellent devices to decrease the risk of vector-borne pathogen transmission, are required. Novel spatial repellent controlled-release devices formulated with metofluthrin were evaluated in an outdoor setting in the northern Amazon of Peru to evaluate performance under field conditions. The metofluthrin emitting devices lowered the number of mosquitoes captured in protected human landing collections (HLC) compared to blank devices, although there were effect differences between *Anopheles* spp. and species in other mosquito genera. A computational-experimental model was developed to correlate HLC and active ingredient (AI) concentrations as a function of time and space. Results show a strong correlation between the released AI and the decrease in HLC. This model represents the first effort to obtain a predictive analytical tool on device performance using HLC as the entomological endpoint.

## 1. Introduction

Mosquito-borne pathogens such as malaria and dengue impact millions of people worldwide [1,2]. U.S. military personnel deployed to regions endemic with vector-borne pathogens are at risk of developing these force-threatening diseases. Because of this threat, the U.S. military has long emphasized personal protection methods including topical repellents such as DEET, which was developed in 1946 by the USDA for the U.S. military [3]. Current personal protection guidance from the U.S. military requires a properly worn permethrin-treated uniform with a topical repellent containing DEET, applied to exposed skin.

Spatial repellents (SRs), such as metofluthrin and transfluthrin, are active ingredients (AIs) designed to elicit spatial mosquito behavioral changes, i.e., causing repellency. Next generation devices are designed to volatilize SRs to create a protective space. Adding an effective spatial repellent, with an effective method of delivery would be an important addition to this guidance, especially during times when uniform discipline is relaxed, and topical repellents are underused. Passive emanators containing metofluthrin in Cambodia demonstrated a landing inhibition rate of *Anopheles* spp. by 48% to 67% depending on the number of emanators used, showing that spatial repellents may play an important role in decreasing human–mosquito contact [4,5,6,7,8,9,10,11].

To find an effective delivery device and spatial repellent active ingredient, the U.S. Naval Medical Research Unit No. 6 (U.S. NAMRU-6) collaborated with GearJump Technologies for a proof-of-concept study of a novel controlled release device in a malaria-endemic region of the northern Peruvian Amazon. Controlled release devices (CRDs) represent the next generation of vector control devices designed to perform controlled release of SRs over prolonged periods of time. Prototype CRDs have been shown in laboratory and semi-field settings to decrease biting and host-seeking behavior for *Anopheles quadrimaculatus* in Florida and *Anopheles gambiae* s.s. in Zambia [12,13]. Unlike several spatial repellent delivery devices currently on the market (e.g., ThermaCell^TM^, coils, candles, etc.), CRDs are battery-less, do not require an external heat source, and can be mass-produced using biodegradable materials at a cost of about USD 0.10 per unit. Once fully tested and optimized, CRDs will be designed to be deployed in both indoor and outdoor settings.

This paper reports the results of a field study performed in an outdoor setting with high mosquito diversity in Mapacocha, Peru [14,15,16], which showed entomological endpoints of CRD efficacy. In addition to measuring entomological outcomes, landing inhibition was correlated to simulated spatial concentrations in the air for a given period for the active ingredient (AI), metofluthrin.

## 2. Materials and Methods

### 2.1. Study Site

The study was conducted in Mapacocha (3°48′58.44″ S; 73°20′28.14″ W), Peru, located along the Nanay River approximately 15 km southwest of the city of Iquitos. The area is rural, consisting of scattered small settlements, agriculture, and secondary growth forest. Currently, *Plasmodium vivax* is the most common malaria species found in villages along the Nanay River and *Anopheles darlingi* is the dominant vector [17]. The mosquito fauna at the study site has been well characterized, with both arbovirus and malaria vector species present year-round [11,12].

### 2.2. Mosquito Collections

**Figure 1:**
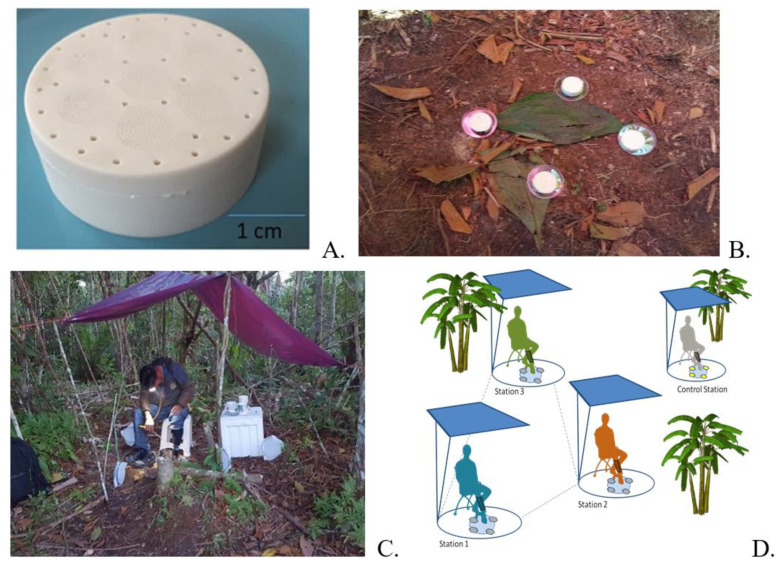
Experimental Setup. (**A**) CRDs. (**B**) Array of CRDs. (**C**) Collection station showing an example of a protected Human Landing Collection. (**D**) Collection Site Block Design.

Mosquitoes were collected using approved protected human landing collections (HLCs) with experienced collectors exposing one stocking-covered leg and using mouth aspirators to collect all mosquitoes that landed within a 6 h sampling period (1800–2400 h), as shown in Figure 1. All collectors were on a physician-prescribed regimen of malaria chemoprophylaxis to prevent malaria infections and wearing protective clothing (Mosquito jacket, long sleeve shirts, long pants). Collections were conducted at the start of each hour for 30 min with a 30 min break. Samples for each site, hour, and treatment were collected in individual cups. Figure 1A shows an image of a CRD. Figure 1B shows the arrangement of 4 CRDs for each location. Figure 1C shows a protected HLC station, showing one collector. Figure 1D shows the layout of the four stations. Three collection sites were used for active devices, and an additional collection site was used for control. Each collector was rotated at the end of each sampling period. The sites were approximately 30 m apart. Collectors were rotated using a randomized block design experiment (M = 3) to reduce bias from one another, for a total of seven rotations (R = 7). The total number of active samples were 21 (N = 21), and the total number of control samples were 6 (N = 6). CRDs were designed for a sustained release of at least one week. To avoid any initial transient response that can lead to performance variability, HLCs were measured six hours after device activation to obtain uniform performance across devices under steady-state conditions [18,19,20].

After collection, mosquitoes were then transported in a cooler to the NAMRU-6 insectary in Iquitos, killed with triethylamine, sorted, and identified morphologically to the lowest possible taxonomic level using dichotomous keys [15]. Voucher specimens for all three experiments are located at the NAMRU-6 Entomology laboratory in Iquitos, Peru.

All experiments were reviewed and approved by the NAMRU-6 Institutional Review Board (Protocol NAMRU6.2016.0003), Dirección Regional de Salud Loreto (DIRESA Loreto) and the Ministerio de Defensa—Ejército del Perú V Division de Ejército. Mosquito collections were performed under the auspices of the Ministerio de Agricultura y Riego del Perú, Dirección General Forestal y de Fauna Silvestre, Resolución Directoral No. 0306-2013-MINAGRI-DGFFS/DGEFFS.

### 2.3. Devices and Active Ingredient

The architecture of the CRDs consists of multiple reservoirs that store 20 mL of the formulated AI. The reservoirs were capped by a permeable membrane and sealed with a pull. Table Metofluthrin was selected as an effective spatial repellent due to its high vapor pressure at room temperature and relatively low toxicity to mammals for dose exposures approved by U.S. Environmental Protection Agency (EPA). While metofluthrin is often considered to be a spatial repellent, the U.S. EPA classifies it as an insecticide [13,21,22,23,24].

CRDs have been designed for long multi-week durations. Optimized formulations of metofluthrin as reported in our previous publications were based on metofluthrin-isopropanol 30% and 100% *v*/*v*. Additional information on CRD design and modelling of metofluthrin dispersion was previously described [12,13].

#### Statistical Analysis

In view of the nonnormality of the HLC findings, nonparametric Wilcoxon and Kruskal–Wallistests are carried out to determine effects due to differences in position, devices and collectors. In addition, Anova and Turkey honest significance parametric tests were carried out on the log transformed HLC results, for which the normality distributed hypothesis cannot be rejected (please refer to Appendix A for further details). Controlled Release Process and Spatial Active Ingredient (AI) Distribution Model.

To address the concentration of AI and the mass that the HLC landing region has, an in silico model is developed to estimate the distributions during the experiments. The simulation was performed to address AI air convection and its effects on the spatial distribution, which resulted in a uniform concentration distribution around a simulated collector. For model details refer to Appendix B.

## 3. Results

Table 1 provides the species of mosquitoes collected, the total number collected by species, and hourly HLC (mosquito/man/hour) across the 11-day experiment. Species collected and abundances were similar to other studies conducted in this area [14,15]. Twenty-eight mosquito species in seven genera were collected in the protected HLCs, including important vector species such as *Anopheles darlingi* and *Culex pedroi*. *Anopheles darlingi* was the most often collected anopheline mosquito (*n* = 455) and *Culex coronator* was the most often collected culicine mosquito (*n* = 1143). Despite the variation in abundance between species, the CRDs were tested against a representation of the mosquito fauna, including *An. darlingi,* the most important malaria vector in the Amazon region.

**Figure 2:**
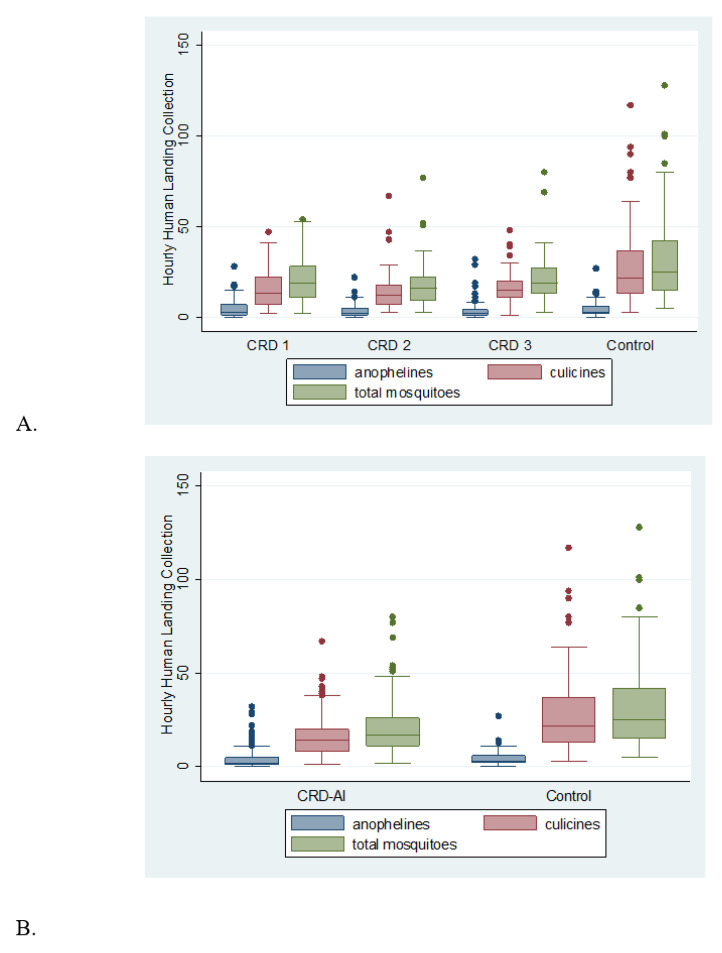
Plots of hourly catches of anophelines, culicines, and total mosquitoes recorded. (**A**) Control and individual CRDs (CRD 1, CRD 2, CRD 3). (**B**) Control CRD versus the mean of number of mosquitoes collected in CRDs with metofluthrin (CRD-AI). Mosquito species were collected using protected human landing collections in Mapacocha, Loreto, Peru, between 10 and 26 January 2017.

An analysis was conducted first to determine if the data were approximately normal. Data were not normally distributed as shown by the tests of Skewness/Kurtosis (*p* < 0.0001), Shapiro–Wilk (*p* < 0.00001) and Shapiro–Francia (*p* < 0.00001). Because we cannot rely on the normal hypothesis, the nonparametric Kruskal–Wallis test was used to determine if mosquito hourly catches differed among devices (CRD 1, CRD 2, CRD 3). Figure 2A shows the HLC distribution among devices. No position effect was found between the four positions, all with *p*-values > 0.05, and there were statistically significant differences between collectors for all cases (anophelines, culicines, and total mosquitoes).

A further non-parametric two-sample Wilcoxon rank-sum test (Mann–Whitney) was applied to determine if untreated control (CRD-C) and combined data from CRDs 1–3 with insecticide (CRD-AI) had an equal impact on the number of mosquitoes caught hourly by protected HLC. In Figure 2B Control and Devices with AI HLCs are shown. Devices with AI had a reductive effect on culicines (CRD-AI = 16.03 vs. CRD-C = 29.2; *p* < 0.0001), and total mosquitoes (CRD-AI = 19.99 vs. CRD-C = 33.39; *p* < 0.0001). The higher variation observed in the control may be attributed to mosquito daily and hourly activity as the field study was carried out with wild mosquitoes. Consistent reduction (smaller variations) of HLC in the active group could be observed regardless of test days and hours. One possible explanation of such a reduced dispersion is that the AI inhibited the overall activity in the active group.

Fewer *Anopheles* spp. were collected in the CRD-AI (*p* = 0.025 for the one-tailed test), which, although not as strong as in the previous cases, is statistically significant as well.

Table 2 shows the number of mosquitoes caught hourly by protected HLC (means and standard deviations) recorded for devices with AI (CDR 1, CRD 2, CRD 3) and untreated control (CRD-C) by mosquito taxonomic group.

Appendix A provides the detailed statistical analysis completed on the log transformed HLC. When applying this transformation, we cannot reject the hypothesis that the data were normal and we therefore could apply all parametric tests (Anova and Turkey honest significance test). Nevertheless, qualitative interpretation of results is cleaner when using the raw data.

## 4. Discussion

This study represents the first test of a novel controlled release technology that also uses an internal exothermic reaction to generate heat to accelerate initial dispersion of a spatial repellent. The novel CRDs tested in an outdoor setting in the Peruvian Amazon decreased the total number of mosquitoes collected in HLCs, including important mosquito vectors, by an average of 30% over the 11 days of the experiment. Despite the high level of variability in the % Landing Inhibition, this proof-of-concept study shows that these CRD devices can decrease human–mosquito contact. However, there was HLC variation between mosquito groups (anophelines and culicines) collected, which is most likely a response to mosquito behavior and environmental variability as has been observed in other field studies [4]. Previous studies investigating the effect of metofluthrin using landing collections in the field reported higher % reductions. Lucas et al. [24,25] found up to 95% reduction for passive paper emanators in Washington State and Florida, USA. Xue et al. [26] in Saint Augustine, FL, found that Off! Clip-on Mosquito Repellent devices (S. C. Johnson and Son, Inc., Racine, WI) with 31% metofluthrin decreased landing rates for *Aedes albopictus* and *Ae. taeniorhynchus* between 70% and 79%, respectively, for three hours. However, this emanator is powered with batteries and actively disperses the AI, which is different from the novel CRDs which use intrinsically produced heat to vaporize the metofluthrin.

Charlwood et al. [4] used passive emanators supplied by Sumitomo Chemical Ltd. (Hyogo, Japan) with 10% metofluthrin, reducing landings in HLCs of *Anopheles* spp. by 48% with just one emanator, and by 67% with four emanators in Cambodia. However, they also found variability in effectiveness between their collection sites with no differences in landing rates between treatments and controls and populations at one site.

A difference between this study, the field evaluations of the Off! Product, and the first field test of Sumitomo emanators, is that we collected mosquitoes over a six-hour period, while the other studies collected over a three-hour period [22], and up to 30 min, respectively [21,27]. Charlwood et al. [4] exposed the emanators to similar conditions to this study and conducted landing collections over 16 days, but only collected for four hours following sunset.

There was no position effect found, however there was a statistically significant difference between collectors in the impact of metofluthrin for *Anopheles* spp. This underscores the importance of testing spatial repellent technologies outside of laboratory and semi-field settings against vector species using the gold standard of mosquito collection for human biting species, the protected HLC.

The use of an experimental–numerical model provided an initial method to predict the efficacy of CRDs by correlation of HLC and simulated spatial AI concentrations for a given period of time. The cumulative AI concentration shows a strong correlation with efficiency or fewer mosquitoes collected in the HLCs. These two observations could be attributed to the fact that the CRDs released more AI in the first day of tests and had a reduced rate for the subsequent days. Hence, the AI released initially contributed to the cumulative efficacy for the remainder of testing period.

The CRDs were tested in realistic outdoor conditions in Amazonian vector-borne endemic areas, and our data show that CRDs provide a platform for potential deployment of SRs outdoors. Additional development and optimization of the CRDs is ongoing and, once finalized, studies measuring CRD efficacy using human health outcomes in addition to entomological endpoints need to be conducted. CRDs with spatial repellents such as metofluthrin or transfluthrin may address an important need in endemic regions where pathogen transmission occurs mainly outdoors, representing a novel technology platform to improve public health.

This field study could be replicated in multiple locations to obtain more comprehensive findings on the use of CRDs as a Public Health tool. Larger field trials, e.g., epidemiological studies involving a greater sample size, would lead to better understanding of the HLC dependence on AI concentration, as well as mosquito resistance.

## 5. Disclaimer

The views expressed in this article reflect the results of research conducted by the authors and do not necessarily reflect the official policy or position of the Department of the Navy, Department of Defense, nor the U.S. Government.

## 6. Copyright Statement

Some authors of this manuscript are military service members and employees of the U.S. Government. This work was prepared as part of their official duties. Title 17 U.S.C. §105 provides that “Copyright protection under this Title is not available for any work of the United States Government”. Title 17 U.S.C. §101 defines a U.S. Government work as a work prepared by a military service member or employee of the U.S. Government as part of that person’s official duties.

## Figures and Tables

**Table 1:** Mosquito species recorded during protected human landing collections in Mapacocha, Loreto, Peru, between 10 and 26 January 2017, evaluating novel spatial repellent devices with metofluthrin. Total number of mosquitoes collected and hourly mosquito catches by species are shown near CRDs with no metofluthrin (Control) and CRDs that released metofluthrin (CRD-AI).

Species	No. Collected	Mosquito/Man/Hour	
		Control	CRD-AI
Anopheline			
*Anopheles* (*Nyssorhynchus*) *benarrochi*	189	0.62	0.75
*Anopheles* (*Nys.*) *darlingi*	455	1.50	1.80
*Anopheles* (*Nys.*) *konderi* s.l.	59	0.23	0.22
*Anopheles* (*Nys.*) sp.	41	0.21	0.14
*Anopheles* (*Nys.*) *triannulatus*	30	0.14	0.11
*Anopheles* (*Anopheles*) *forattinii*	109	0.42	0.41
*Anopheles* (*Ano.*) *peryassui*	177	1.06	0.54
Culicine			
*Aedes* (*Ochlerotatus*) *fulvus*	352	1.24	1.36
*Aedes* (*Och.*) *serratus*	419	1.85	1.50
*Coquillettidia* (*Rhychotaenia*) *venezuelensis*	471	2.06	1.69
*Coquillettidia* (*Rhy.*) *nigricans*	9	-	0.05
*Coquillettidia* spp.	4	0.03	0.01
*Culex* (*Culex*) *coronator*	1143	8.42	2.96
*Culex* (*Cux.*) *quinquefasciatus*	23	0.03	0.11
*Culex* (*Melanoconium*) *gnomatus*	8	0.08	0.02
*Culex* (*Mel.*) *ocossa*	3	0.02	0.01
*Culex* (*Mel.) pedroi*	121	1.00	0.28
*Culex* (*Mel.*) *portesi*	3	0.03	0.01
*Culex* (*Mel.*) sp. 1	58	0.45	0.14
*Culex* (*Mel.*) *spissipes*	9	0.09	0.02
*Culex* (*Mel.*) *theobaldi*	768	4.65	2.33
*Culex* (*Mel.*) *vomerifer*	24	0.21	0.05
*Johnbelkinia longipes*	1	0.02	-
*Mansonia* (*Mansonia.*) *indubitans/titillans*	233	1.48	0.68
*Mansonia* (*Man.*) *humeralis*	1	-	0.01
*Psorophora* (*Grabhania*) *cingulata*	689	3.36	2.36
*Psorophora* (*Janthinosoma*) *albigenu*	668	3.48	2.21
*Psorophora* (*Janthinosoma*) *ferox*	95	0.70	0.25
Total	6162	33.39	19.99

Average temperatures from 10 to 26 January 2017 ranged between 24.5 °C min and 30.5 °C max, and average relative humidity ranged between 67.2% min and 85.7% max. There was variation in the number of mosquitoes collected each sampling day, and variation in the mean % landing inhibition for total mosquitoes collected that ranged from −55% to 76% with a mean % landing inhibition over the 11 days of 30%.

**Table 2:** Hourly human landing collections (Means ± SD) recorded for devices with insecticide (CRD 1, CRD 2, CRD 3) and with no metofluthrin (Control) by mosquito taxonomic group.

Taxonomic Group	CRD 1	CRD 2	CRD 3	Control
Anophelinae	4.83 ± 5.32	3.15 ± 4.04	3.89 ± 6.16	4.18 ± 4.22
Culicinae	16.03 ± 10.49	14.82 ± 11.53	17.24 ± 9.82	29.21 ± 23.77
Total Mosquitoes	20.86 ± 12.34	17.97 ± 13.04	21.14 ± 13.27	33.39 ± 25.43

**Figure A1:**
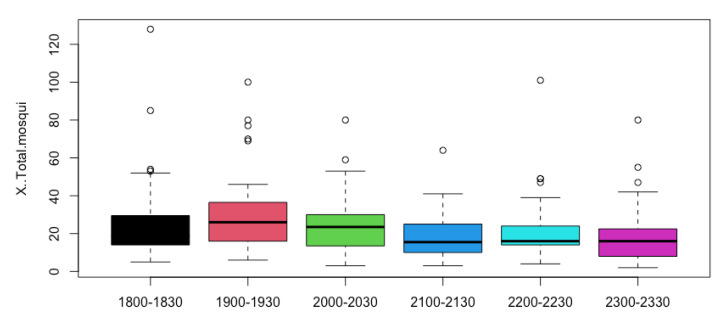
Number of captures for given time period.

**Figure A2:**
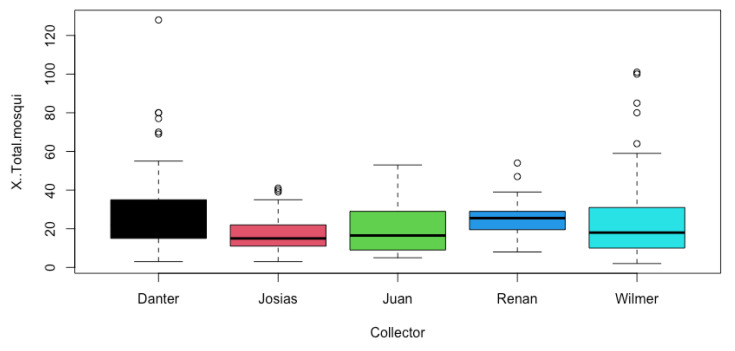
Mosquito captures per collector.

**Figure A3:**
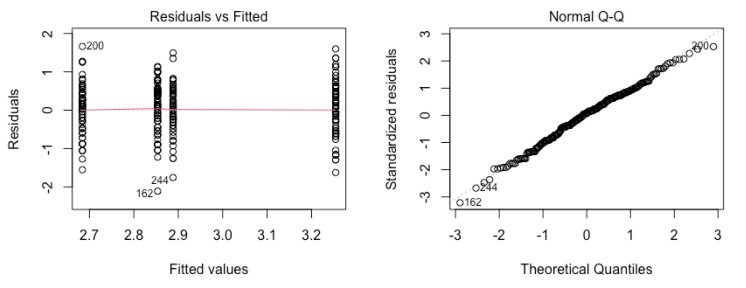
Error Distribution Analysis.

**Figure A4:**
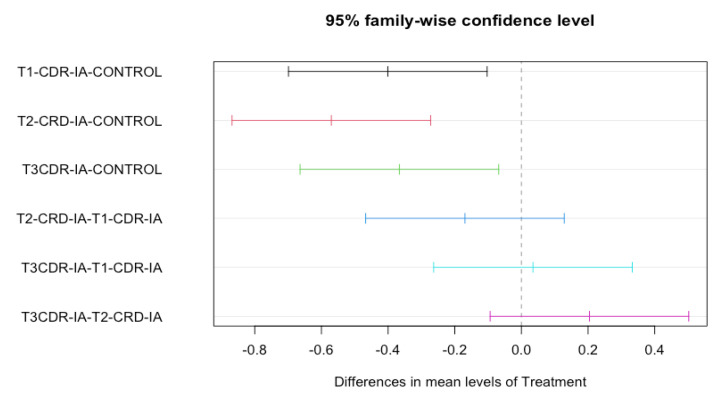
Differences in mean levels of treatment.

**Figure A5:**
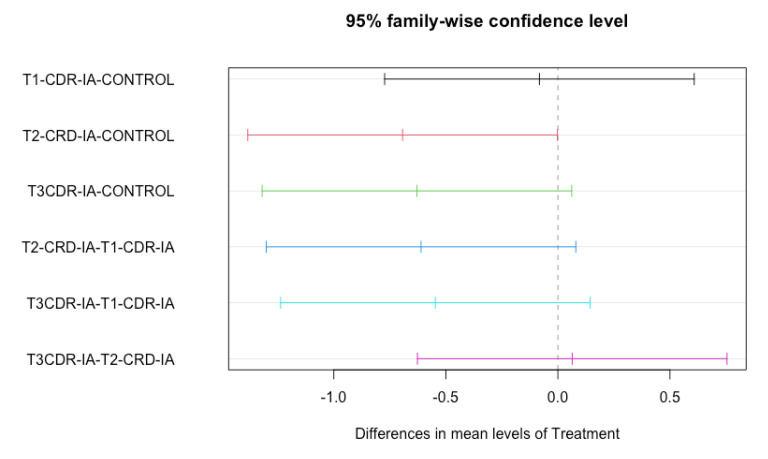
Confidence Intervals for Tukey Test for anophelines.

**Figure A6:**
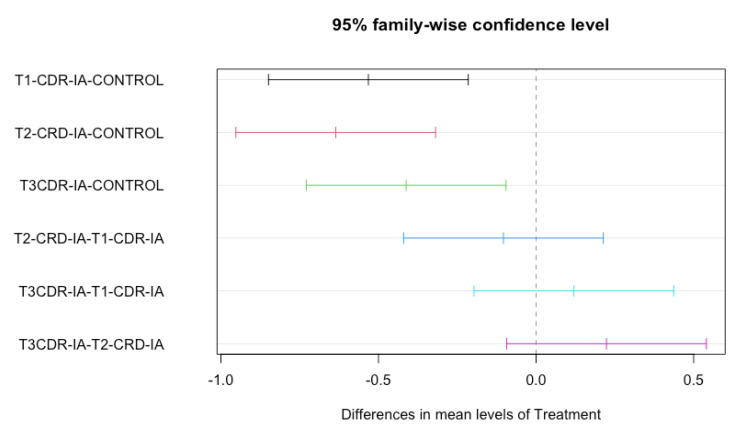
Confidence Intervals for Tukey Test for culicines.

**Figure A7:**
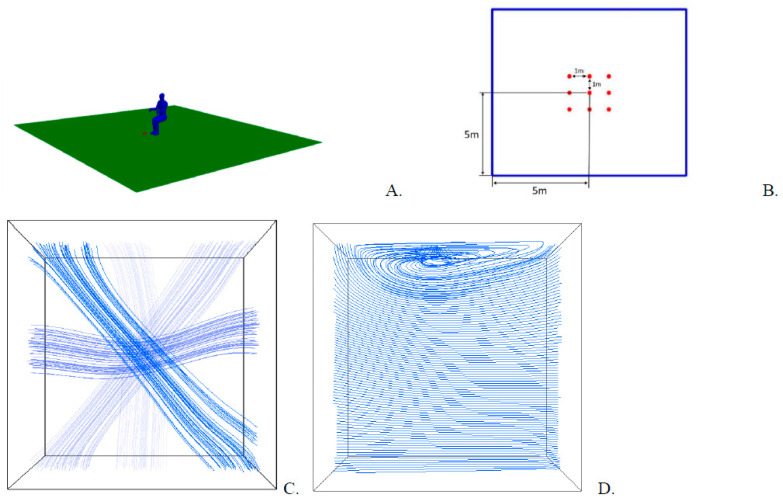
Domain and currents. (**A**) Domain used for simulations. (**B**) Subject location inside the study domain. (**C**) Currents crossing the domain. (**D**) Currents creating vortices.

**Figure A8:**
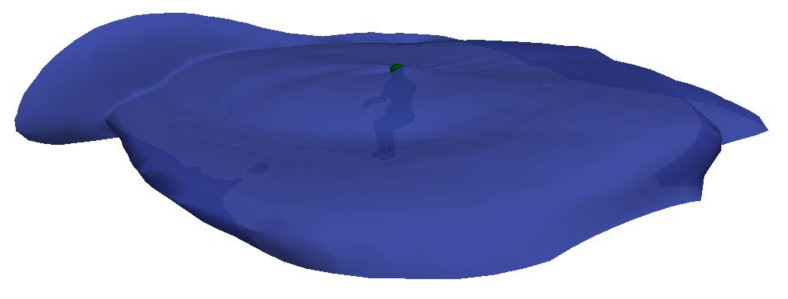
Simulated distribution. Isosurface using concentration envelope of 1 µg/m^3^ for device mass rate of 6.444 µg/s.

**Figure A9:**
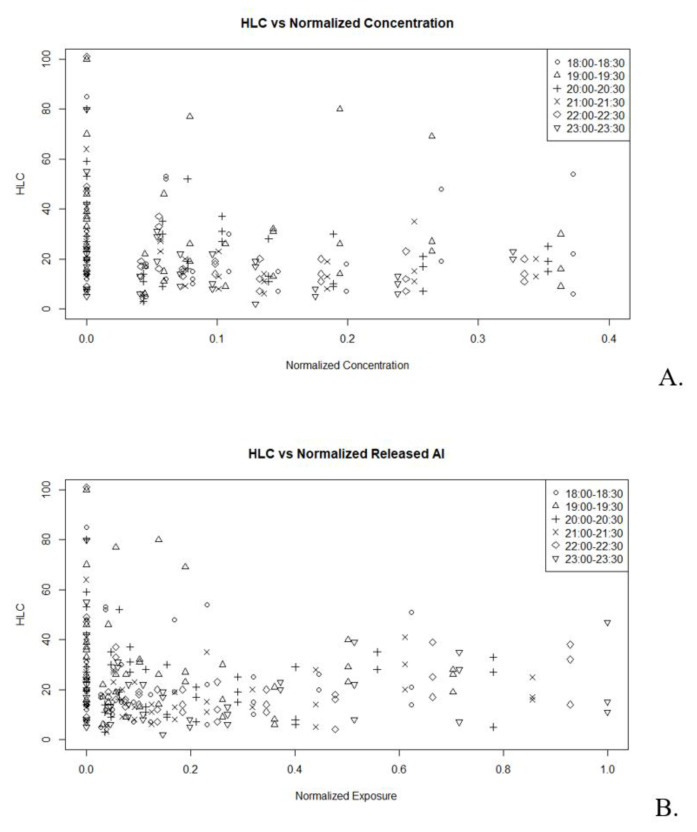
Simulated concentration and mass released. (**A**) HLC vs. normalized concentration. (**B**) HLC vs. normalized released AI.

## Data Availability

Data generated or analyzed during this study are included in this published article.

## Appendix A

### Appendix A.1. Introduction

The objective of this analysis is to further statistically assess the effect of a spatial repellent against mosquitoes. The Dataset consists of 264 experimental units, measured within 11 days of field work. We have the following important data aspects to take into account:

- Four measurement stations (separating distances are negligible);
- Five human collectors;
- Six consecutive measurement time periods of 30 min each day;
- Four treatments: three with repellent (same doses) and a control;
- HLC broken down by subfamily (Anophelinae and Culicinae).

We define the experimental Unit to be the HLC in a specific (Time Slot, Collector, Station, Treatment) tuple.

### Appendix A.2. Data Description

We compute several statistics comparing the cases grouped by Treatment and Control, shown in Table A1.

**Table A1:** Comparison among treatments.

Control	Mean	Median	sd	n
Control	33.39	25	25.43	66
Repellent	19.99	17	12.90	198

The percent drop when the repellent is present is of 40% (mean) and 32% (median). We stress that 25% of the experimental units were assigned to control, whereas the remaining 75% were assigned to Treatment with Repellent (i.e., 198 units).

This comparison may be encouraging, but before assessing the effect of repellents we need to investigate the influence of additional factors on the number of captured specimens. We describe below some of those factors in detail.

#### Appendix A.2.1. Time of Day Condition

Every day, data were collected during the following times:

- 1800–1830
- 1900–1930
- 2000–2030
- 2100–2130
- 2200–2230
- 2300–2330

It is a well-known fact that mosquitoes tend to reduce their activity during the late hours of the evening. We studied the effect of this variable on the number of captures and found this effect to be present in the analysis. Results are plotted in Figure A1 and some basic stats are displayed below in Table A2.

**Table A2:** Basic Statistics by Hour.

Hour	Mean	Median	sd	Obs
1800–1830	26.18	19.0	22.81	44
1900–1930	30.05	26.0	20.98	44
2000–2030	24.59	23.5	15.46	44
2100–2130	18.45	15.5	11.79	44
2200–2230	21.75	16.0	16.48	44
2300–2330	19.02	16.0	15.34	44

We stress that the four treatments are uniformly distributed among the different hours of the day. There were 11 observations per each time period per station. There is therefore no bias in the assignment for this factor.

#### Appendix A.2.2. Collectors

Biological factors of the Collector, such as sweat, may bias the amount of Mosquitoes collected. For this purpose, we derive some basic descriptive statistics for the 5 different collectors (shown by name), shown in Figure A2 and Table A3.

**Table A3:** Basic Statistics by Collector.

Collector	Mean	Median	Std Dev	Obs
Danter	28.65	22.0	22.29	66
Josias	17.30	15.0	9.09	66
Juan	20.50	16.5	13.20	42
Renan	26.04	25.5	10.70	24
Wilmer	24.89	18.0	21.96	66

The distribution of Collectors across the different treatments, Table A4, was not uniform. Given the natural differences among collectors and logistical challenges, and the environmental changes, it was extremely difficult to obtain a uniform distribution in collection hours, which complicates the Treatment–Collector interaction analysis.

**Table A4:** Distribution of Observations by Collector and Treatment.

	Danter	Josias	Juan	Renan	Wilmer
CONTROL	24	0	24	0	18
T1-CDR-IA	0	0	18	24	24
T2-CRD-IA	18	24	0	0	24
T3CDR-IA	24	42	0	0	0

#### Appendix A.2.3. Position (Station)

The four stations do not exhibit relevant difference in medians (which remove the distorting effect of outliers). The GPS locations of those are quite near each other so this is expected, as shown in Table A5. We therefore do not include the variable “position” in subsequent analysis.

**Table A5:** Statistics by Position.

Position	Mean	Median	n
1	22.94	19	66
2	23.56	20	66
3	19.79	18	66
4	27.08	19	66

#### Appendix A.2.4. Mosquito Subfamilies

Two subfamilies of mosquitoes were analyzed: Anophelinae and Culicinae. Given the importance of each subfamily, we repeated the analysis for each subfamily as well as for the combined number of mosquitoes.

### Appendix A.3. Effect of Repellent

The first step in our analysis was to Log transform HLC. This helped to mitigate outliers and resulted in better error distributions during the analysis. First, we measured the standard deviation for each treatment, shown in Table A6.

**Table A6:** Standard deviation per treatment.

Treatment	Std Dev
CONTROL	0.73
T1-CDR-IA	0.66
T2-CRD-IA	0.65
T3CDR-IA	0.60

The standard deviations have small differences and justify the ANOVA analysis below to assess the effects across treatments.

#### Appendix A.3.1. ANOVA

We first check if there are statistical differences across Treatments. The ANOVA model we tested was:

$$y_{i,j}=\mu +\tau_{i}+\epsilon_{i,j}$$

where:

- $y_{i,j}$: log HLC of experimental Unit “*j*” for Treatment “*i*”;
- μ: Global Mean of log HLC;
- $\tau_{i}$: Mean of log HLC’s for treatment “*i*”;
- $\epsilon_{i,j}$: Error of experimental Unit “*j*” in Treatment “*i*”.

The results are displayed in Table A7.

**Table A7:** Summary of ANOVA analysis.

	Degrees of Freedom	Sum of Squares	Mean of Sum of Squares	F Value	PR (>F)
Treatment	3	11.41	3.803	8.678	1.65 × 10^−6^ ***
Residuals	260	113.95	0.438		

Explanation of Reference *p* Values Thresholds: ***, 0.001.

We see that differences between Treatments cannot be neglected. The F and *p* values are quite significant (*p* value = 1.65 × 10^−5^). Error checking is necessary at this stage, and we obtain reasonable results. No patterns are visible in the errors and the distribution approaches normality quite closely, as shown in Figure A3.

#### Appendix A.3.2. Comparison of Treatments Effects

We now take a look at the predicted levels for each Treatment, Table A8.

**Table A8:** Predicted Treatment Levels (ANOVA Analysis).

CONTROL	T1-CRD-IA	T2-CRD-IA	T3-CRD-IA
0.3343	−0.0665	−0.2360	−0.0318

We realize that the Control Treatment does exhibit the largest (and only positive) difference in log HLC against the normalized global mean of the treatments (0). The log HLC for all other treatments (with dose), are moderately or markedly below the global mean.

To better understand the effects on an individual level for each treatment we perform a Pairwise Tukey Test with a confidence level of 95% and derive the corresponding confidence intervals, shown in Figure A4.

The Confidence Intervals displayed above correspond to the six possible differences between the four different Treatments. It is interesting to note that the three upper confidence intervals (representing all three differences in Log HLC between dose and control) are all centered on negative values and do not contain the zero. In other words, for a 95% confidence interval, we expect the dose to be below control. The remaining three lower confidence intervals include the zero value and are roughly centered around it, which points to negligible differences across treatments with different doses. It is worth mentioning that the Tukey test is more stringent and therefore places higher demands on the data to reject the null hypothesis. This renders the results even more meaningful.

#### Appendix A.3.3. Interaction Effects

To statistically assess any possible interaction effects, we fitted a 2-factor model (Treatment * Hour) with interaction (in fact, Hour is a Block or nuisance factor). The idea is to measure the individual and combined effects. In case there is some interaction, we have reasons to suspect that the Log HLC was influenced by the Hour. The model used was:

$$y_{i,j,k}=\mu +\tau_{i}+\beta_{j}+\tau \beta_{i,j}+\epsilon_{i,j,k}$$

where:

- $y_{i,j,k}$: Log HLC within experimental unit “*k*” for Treatment “*i*” and Hour “*j*”;
- μ: Global Mean of Log HLC;
- $\tau_{i}$: Effect of treatment “*i*” on Log HLC;
- $\beta_{j}$: Effect of Block “*j*” on Log HLC;
- $\tau \beta_{i,j}$: Effect of interaction of Treatment “*i*” and Hour “*j*” on Log HLC;
- $\epsilon_{i,j,k}$: Error of experimental unit “*k*” in Treatment “*i*” and Hour “*j*” on Log HLC.

The results are displayed in Table A9.

**Table A9:** Interaction Results.

	Degrees of Freedom	Sum of Squares	Mean of Sum of Squares	F Value	PR (>F)
Treatment	3	11.41	3.803	9.114	9.79 × 10^−6^ ***
Hour	5	8.12	1.624	3.892	0.00207 **
Treatment:Hour	15	5.67	0.378	0.906	0.55818
Residuals	240	100.15	0.417		

Explanation of Reference *p* Values Thresholds: ***, 0.001; **, 0.01.

We realize that the Blocking factor (Hour) is significant to explain differences in Log HLC, although to a weaker extent. In parallel, we did not obtain evidence for interaction between Treatment and Hour (*p* value = 0.56). In summary, even though the time of day influenced Log HLC, this was weaker than the treatment effect and both Treatment and Hour are not related, which further supports the evidence found in Section 3 that the repellent had a significant effect on reducing Log HLC.

A similar analysis should be run to assess the interaction between the Collector and the Treatment (with Collector as a Block factor); the samples were quite unbalanced, with multiple cases presenting the complete absence of a collector from a treatment type. We should defer this specific case to a later stage when more favorable data is available. Nevertheless, we can partially investigate those cases of Collectors which show relatively balanced data. Selecting three collectors and assuming that two treatments with repellent are roughly the same, we test a new model investigating the interaction between Treatment and Collector, as shown in Table A10.

**Table A10:** Collector-Treatment Analysis.

	Degrees of Freedom	Sum of Squares	Mean of Sum of Squares	F Value	PR (>F)
Treatment	1	7.11	7.113	16.273	9.45 × 10^−5^ ***
Hour	2	4.36	2.178	4.982	0.00827 **
Treatment:Collector	2	5.19	2.597	5.942	0.00342 **
Residuals	126	55.08	0.437		

Explanation of Reference *p* Values Thresholds: ***, 0.001; **, 0.01.

As we can see, the most significant effect is the treatment, followed by the interaction term, which in this case cannot be neglected at the 1%. We need to acknowledge that this result may be biased but should be investigated further in the future when the experiment is improved to avoid undesirable biases.

### Appendix A.4. Analysis by Subfamily

As mentioned earlier, there are two subfamilies of mosquitoes. In this section, we take a closer look at the response to repellents by subfamily of mosquito. For that purpose, we break down the total number of mosquitoes captured in each experimental unit in the two groups: Anophelinae and Culicinae mosquitoes analyze each, one at a time.

#### Appendix A.4.1. Anophelinae

In the case of anophelines, the presence of a repellent seems to make a difference in the Log HLC at a 5% confidence, but not at 1%. Table A11 shows the Anova summary.

**Table A11:** Summary of Anova analysis for anophelines.

	Degrees of Freedom	Sum of Squares	Mean of Sum of Squares	F Value	PR (>F)
Treatment	3	25.7	8.574	3.641	0.0133 *
Residuals	260	612.3	2.355		

Explanation of Reference *p* Values Thresholds: *, 0.05.

When analyzing the confidence intervals, we realize that the pairwise differences is weaker than for the case of total mosquitoes and all intervals include the zero at a 95% confidence, as shown in Figure A5.

#### Appendix A.4.2. Culicinae

In the case of culicines, the presence of a repellent seems to make a stronger difference, as shown in Table A12 and Figure A6.

**Table A12:** Summary of Anova analysis for culicines.

	Degrees of Freedom	Sum of Squares	Mean of Sum of Squares	F Value	PR (>F)
Treatment	3	15.38	5.126	10.35	1.87 × 10^−6^ ***
Residuals	260	128.82	0.495		

Explanation of Reference *p* Values Thresholds: ***, 0.001.

When analyzing the confidence intervals, the pairwise differences against Control are quite significant and none include the zero at a 95% confidence.

## Appendix B

### Appendix B.1. Controlled Release Process and Spatial Active Ingredient (AI) Distribution Model

AI released from a concentrated source in air is driven by a convection–diffusion mass transport process. When dealing with low diffusivity molecules such as metofluthrin (D = 6.8 × 10^−6^ m^2^/s), the diffusion is negligible when compared with convection. This means that the molecules are advected by the air movement and the diffusion has little or no effect. Consequently, AI distribution is strongly conditioned by air movement.

In open spaces, random wind currents cannot be easily measured and frequently the only available parameter is average wind speed. In the numerical model, a series of velocity fields that preserved the average wind speed were applied. These distributions were defined as harmonic oscillations with smooth speed variations in order to simulate natural convected air flow in the defined domain. Two kind of air currents were considered: (1) currents crossing the domain; (2) currents creating vortices in the domain.

### Appendix B.2. Currents Crossing the Domain

The velocity components aligned with wind mean velocity for winds crossing the domain were defined as follows:

$$\begin{array}{c} V_{x}=V_{a}\sin\left(2\pi ft\right)+V_{m}\\ V_{y}=V_{a}\sin\left(2\pi ft+\frac{\pi }{2}\right)\\ V_{z}=V_{a}\sin\left(2\pi \frac{f}{3}t\right) \end{array}$$

where *V_a_* is the amplitude velocity, *V_m_* is the mean velocity and *f* the frequency (wind oscillation). Note that averages in time *V_x_ = V_m_*, and *V_y_ = V_z_* = 0, and that the vertical component frequency is 1/3 of the horizontal ones, to address that the wind affects directly horizontal components, but has less effect in the vertical component.

### Appendix B.3. Currents Creating Vortices in the Domain

The velocity components, applied tangentially to the domain opposite walls for winds creating vortices in the domain were defined as follows:

$$\begin{array}{c} V_{x}^{+}=-V_{x}^{-}=V_{a}\sin\left(2\pi ft\right)\\ V_{y}^{+}=-V_{y}^{-}=V_{a}\sin\left(2\pi ft+\pi \right)\\ V_{z}=V_{a}\sin\left(2\pi \frac{f}{3}t\right) \end{array}$$

where $V_{x}^{+}\;and\;V_{x}^{-}$ are the velocities applied to opposite domain faces.

Considering low velocity air conditions (Beaufort scale), the mean velocity was set to *V_m_* = 1.11 m/s and amplitude velocity to *V_a_ = V_m_*, 2 *V_m_* and 3 *V_m_*. Frequency values were defined as *f* = 0.05 Hz, 0.025 Hz and 0.0125 Hz, whereas vertical speed *V_z_* = 0.0555 m/s, 0.111 m/s and 0.222 m/s. All the proposed air currents were simulated (Open FOAM) [28].

Once the velocity fields were defined, the mass transport process based on convection was simulated for nine different locations of the CRDs. For each proposed wind condition, nine subject locations inside the simulation domain were evaluated. Mass device release rate was set initially to 6.444 µg/s and reduced daily, according to data obtained by characterization of the device depletion. All simulated wind cases per location were averaged to determine the mean concentration found for each point in space.

### Appendix B.4. Spatial AI Distribution Model

The domain analyzed is shown in Figure A7, subject locations inside the domain in Figure A7B and stream-lines for both currents crossing the domain and currents creating vortices in the Domain are provided in Figure A7C,D.

Regarding AI coverage, from the simulations it can be observed that the expected AI distribution reaches all the study area consistently. In Figure A8 we provide the isosurface that encloses the spatial envelope with concentrations higher than 1 µg/m^3^.

To analyze simulations, HLC is related to the expected AI normalized concentration (relative to maximum achieved along the experiments) and to normalized released AI (relative to maximum released per night). Figure A9 shows experimental simulation results showing HLC vs. normalized concentration HLC, and in Figure A9A and HLC vs. normalized released AI in Figure A9B for different hours to address mosquito pressure effect. For high mosquito pressure, a reduction in HLC can be observed for both indicators.

## References

[1] Ashley E.A., Pyae Phyo A., Woodrow C.J. (2018). Malaria. Lancet.

[2] Bhatt S., Gething P.W., Brady O.J., Messina J.P., Farlow A.W., Moyes C.L., Drake J.M., Brownstein J.S., Hoen A.G., Sankoh O. (2013). The global distribution and burden of dengue. Nature.

[3] Debboun M., Strickman D.A., Klun J.A. (2005). Repellents and the military: Our first line of defense. J. Am. Mosq. Control Assoc..

[4] Charlwood J.D., Nenhep S., Protopopoff N., Sovannaroth S., Morgan J.C., Hemingway J. (2016). Effects of the spatial repellent metofluthrin on landing rates of outdoor biting anophelines in Cambodia, Southeast Asia. Med. Vet. Entomol..

[5] Achee N.L., Bangs M.J., Farlow R., Killeen G.F., Lindsay S., Logan J.G., Zwiebel L.J. (2012). Spatial repellents: From discovery and development to evidence-based validation. Malar. J..

[6] Syafruddin D., Asih P.B., Rozi I.E., Permana D.H., Nur Hidayati A.P., Syahrani L., Liu F. (2020). Efficacy of a Spatial Repellent for Control of Malaria in Indonesia: A Cluster-Randomized Controlled Trial. Am. J. Trop. Med. Hyg..

[7] Syafruddin D., Bangs M.J., Sidik D., Elyazar I., Asih P.B., Chan K., Ishak H. (2014). Impact of a spatial repellent on malaria incidence in two villages in Sumba, Indonesia. Am. J. Trop. Med. Hyg..

[8] Hill N., Zhou H.N., Wang P., Guo X., Carneiro I., Moore S.J. (2014). A household randomized, controlled trial of the efficacy of 0.03% transfluthrin coils alone and in combination with long-lasting insecticidal nets on the incidence of Plasmodium falciparum and Plasmodium vivax malaria in Western Yunnan Province, China. Malar. J..

[9] Khater E., Zhu D., Bibbs C., Xue R., Peper S. (2021). Insecticide efficacy of spatial repellent compound-metofluthrin against susceptible and resistant strains of aedes aegypti. J. Fla. Mosq. Control Assoc..

[10] Permana D.H., Zubaidah S., Syahrani L., Asih P.B.S., Syafruddin D., Rozi I.E., Hidayati A.P.N., Kosasih S., Dewayanti F.K., Rachmawati N. (2022). Impact of a spatial repellent product on Anopheles and non-Anopheles mosquitoes in Sumba, Indonesia. Malar. J..

[11] Ochomo E.O., Gimnig J.E., Bhattarai A., Samuels A.M., Kariuki S., Okello G., Abong’O B., Ouma E.A., Kosgei J., Munga S. (2022). Evaluation of the protective efficacy of a spatial repellent to reduce malaria incidence in children in western Kenya compared to placebo: Study protocol for a cluster-randomized double-blinded control trial (the AEGIS program). Trials.

[12] Bernier U.R., Kline D.L., Vazquez-Abad A., Perry M., Cohnstaedt L.W., Gurman P., D’Hers S., Elman N.M. (2019). A combined experimental-computational approach for spatial protection efficacy assessment of controlled release devices against mosquitoes (*Anopheles*). PLoS Neglected Trop. Dis..

[13] Stevenson J.C., Simubali L., Mudenda T., Cardol E., Bernier U.R., Vazquez A.A., Thuma P.E., Norris D., Perry M., Kline D.L. (2018). Controlled release spatial repellent devices (CRDs) as novel tools against malaria transmission: A semi-field study in Macha, Zambia. Malar. J..

[14] Need J.T., Rogers E.J., Phillips I.A., Falcon R., Fernandez R., Carbajal F., Quintana J. (1993). Mosquitoes (Diptera: Culicidae) Captured in the Iquitos Area of Peru. J. Med. Entomol..

[15] Peck G.W., Castro-Llanos F., López-Sifuentes V.M., Vásquez G.M., Lindroth E. (2018). Comparative Analysis of Mosquito Trap Counts In the Peruvian Amazon: Effect of Trap Type and Other Covariates On Counts and Diversity. J. Am. Mosq. Control Assoc..

[16] Morrison A.C., Reiner R.C., Elson W.H., Astete H., Guevara C., Del Aguila C., Bazan I., Siles C., Barrera P., Kawiecki A.B. (2022). Efficacy of a spatial repellent for control of Aedes -borne virus transmission: A cluster-randomized trial in Iquitos, Peru. Proc. Natl. Acad. Sci. USA.

[17] Moreno M., Saavedra M.P., Bickersmith S.A., Lainhart W., Tong C., Alava F., Vinetz J.M., Conn J.E. (2015). Implications for changes in Anopheles darlingi biting behaviour in three communities in the peri-Iquitos region of Amazonian Peru. Malar. J..

[18] Dame D.A., Meisch M.V., Lewis C.N., Kline D.L., Clark G.G. (2014). Field Evaluation of Four Spatial Repellent Devices Against Arkansas Rice-Land Mosquitoes. J. Am. Mosq. Control Assoc..

[19] Ogoma S.B., Moore S.J., Maia M.F. (2012). A systematic review of mosquito coils and passive emanators: Defining recommendations for spatial repellency testing methodologies. Parasites Vectors.

[20] WHO (2013). Guidelines for Efficacy Testing of Spatial Repellents.

[21] Buhagiar T.S., Devine G.J., Ritchie S.A. (2017). Effects of sublethal exposure to metofluthrin on the fitness of Aedes aegypti in a domestic setting in Cairns, Queensland. Parasites Vectors.

[22] Bibbs C.S., Kline J., Kline D.L., Estaver J., Strohschein R., Allan S.A., Batich C.D. (2020). Olfactometric comparison of the volatile insecticide, metofluthrin, through behavioral responses of Aedes albopictus (Diptera: Culicidae). J. Med. Entomol..

[23] Ujihara K., Mori T., Iwasaki T., Sugano M., Shono Y., Matsuo N. (2004). Metofluthrin: A Potent New Synthetic Pyrethroid with High Vapor Activity against Mosquitoes. Biosci. Biotechnol. Biochem..

[24] Lucas J.R., Shono Y., Iwasaki T., Ishiwatari T., Spero N. Field Efficacy of Metofluthrin—A New Mosquito Repellent. Proceedings of the Fifth International Conference on Urban Pests.

[25] Lucas J.R., Shono Y., Iwasaki T., Ishiwatari T., Spero N., Benzon G.U.S. (2007). Laboratory and field trials of metofluthrin (SumiOne^®^) emanators for reducing mosquito biting outdoors. J. Am. Mosq. Control Assoc..

[26] Xue R.D., Qualls W.A., Smith M.L., Gaines M.K., Weaver J.H., Debboun M. (2012). Field evaluation of the Off! Clip-on Mosquito Repellent (metofluthrin) against Aedes albopictus and Aedes taeniorhynchus (Diptera: Culicidae) in northeastern Florida. J. Med. Entomol..

[27] Achee N., Masuoka P., Smith P., Martin N., Chareonviryiphap T., Polsomboon S., Grieco J. (2012). Identifying the effective concentration for spatial repellency of the dengue vector Aedes aegypti. Parasites Vectors.

[28] Weller H.G., Tabor G., Jasak H., Fureby C. (1998). A tensorial approach to computational continuum mechanics using object-oriented techniques. Comput. Phys..

